# Return to work with long COVID: a rapid review of support and challenges

**DOI:** 10.1136/bmjopen-2025-101698

**Published:** 2025-10-07

**Authors:** Sarah Daniels, Hua Wei, Damien M McElvenny, Martie van Tongeren, Donna Bramwell, Anna Coleman, Davine Forde, Ruth Wiggans

**Affiliations:** 1 Division of Population Health, Health Sciences Research and Primary Care, The University of Manchester, Manchester, UK; 2 Institute of Occupational Medicine, Edinburgh, UK; 3 National Institute for Health and Care Research (NIHR) Manchester Biomedical Research Centre, Manchester, UK; 4 National Institute for Health and Care Research (NIHR) Manchester Clinical Research Facility, Manchester, UK; 5 Department of Respiratory Medicine, North Manchester General Hospital, Manchester, UK

**Keywords:** Long COVID, Return to work, Workplace support, Employment, Occupational health

## Abstract

**Abstract:**

**Objectives:**

To explore existing evidence for the provision of support for return to work (RTW) in long COVID (LC) patients and the barriers and facilitators to taking up this support.

**Design:**

A rapid review reported in accordance with the Preferred Reporting Items for Systematic Reviews and Meta-Analyses guidelines. The study was preregistered in PROSPERO (ID: CRD42023478126).

**Data sources:**

Searches were completed in June 2024 across major databases including MEDLINE, Embase, PsycINFO, evidence-based medicine reviews, Web of Science and Google Scholar.

**Eligibility criteria:**

Included studies focused on people with LC (PwLC) symptoms lasting over 12 weeks and addressed either: (1) non-workplace- or workplace-based support for RTW and/or (2) barriers and facilitators to RTW in this population.

**Data extraction and synthesis:**

A quality assessment was conducted using the JBI Systematic Reviews critical appraisal tool. The data were summarised in tabular format and a narrative synthesis.

**Results:**

Twenty-five studies were included. While many studies demonstrated rigorous methodologies and low risk of bias levels, some had high and medium risk levels. Non-workplace-based support was mostly measured quantitatively and included interdisciplinary healthcare programmes, clinical interventions and rehabilitation programmes focusing on pacing and breathing strategies. Compensation and insurance schemes were important funders of these interventions.

Workplace-based support was mostly measured qualitatively. Barriers to the provision of support at organisational level included lack of understanding of LC symptoms, insufficient workplace guidance and educational gaps among managers. Individual barriers included threat of income loss, remote working and disconnection from the workplace. Facilitators for support included recognition and validation of LC and its symptoms, and eligibility for disability benefits associated with work.

**Conclusions:**

RTW is an important outcome of health-related absence and should be systematically recorded in studies of PwLC. The heterogeneity and unpredictability of LC symptoms create challenges for supporting working age populations. Further research is crucial to better understand the specific RTW needs for PwLC and address potential barriers and facilitators to workplace-based support, particularly through interventions, organisational practices and employ-led policies that enable sustained RTW. Consistent guidelines on LC’s definition and disability status may facilitate the provision of support and the development of interventions.

**Prospero registration number:**

CRD42023478126.

**STRENGTHS AND LIMITATIONS OF THIS STUDY**

- Offers an in-depth examination of workplace and non-workplace-based support for return to work (RTW) for patients with long COVID (LC), including clinical interventions, multidisciplinary rehabilitation programmes, workplace support and self-developed strategies.
- Comprehensive literature search of major electronic databases across disciplines and reporting as per Preferred Reporting Items for Systematic Reviews and Meta-Analyses guidelines.
- The search was limited to English language, likely excluding relevant studies.
- While many studies had a low risk of bias, some studies had selection bias, unvalidated outcome measures or lacked control of confounders, limiting result accuracy.
- The focus on work-related outcomes excluded alternative support measures that might impact RTW, and the strict definition of LC omitted studies on overlapping conditions like chronic fatigue syndrome.

## Introduction

 Long COVID (LC) is a multisystem condition with symptoms persisting for 12 weeks (also known as post-COVID-19 syndrome, PSC).^1^ Common symptoms include chronic fatigue, shortness of breath, difficulty concentrating and muscle aches.^2^

Globally, LC affects over 400 million people and remains a complex, heterogeneous condition with substantial socioeconomic impacts.^3^ The 2025 Delphi consensus study involving healthcare professionals, researchers and individuals with lived experience established comprehensive consensus on the need for clearer definitions, diagnostic criteria and treatment pathways and more research into the social and economic burden of LC.^3^ This complements earlier perspectives highlighting the episodic nature of LC symptoms, the ongoing transmission of SARS-CoV-2 variants and the insufficiency of current healthcare responses to address the growing burden of LC.^4^

In the UK, approximately 2 million people (3.3% of the population) in England and Scotland were experiencing self-reported LC as of March 2024.^5^ LC was more prevalent in middle age (aged 45 to 64), females and in those who were not working and not looking for work. Some symptoms can be quite severe and would prevent a person from trying to return to work (RTW).^6^

Supporting workers living with long-term conditions such as LC to RTW is important because employment benefits both physical and mental health, providing financial security and fulfilling psychosocial needs.^7^ Unemployment is linked to higher mortality rates and poorer health outcomes.^7 8^ One study found that 45% of people with LC (PwLC) had to reduce their work schedules and 22% were not working 7 months after symptoms began.^9^

Previous knowledge on RTW after long-term sickness absence may not be easily translatable to PwLC.^10^ Compared with other conditions, LC symptoms often fluctuate, complicating a linear recovery process and making existing RTW measures less applicable.^10^ LC is experienced differently across individuals, inconsistently defined in the literature and variously interpreted in practice. As a result, individuals’ experiences of the condition, their diagnoses, treatment and the impacts on their life may also vary substantially. This has also changed as understanding of the condition has evolved, so PwLC diagnosed in 2020 may have had different experiences to someone diagnosed in 2025. Existing policies, especially those related to absence management, may not be adequate to meet the needs of PwLC.^11^ The availability and effectiveness of support for RTW for PwLC are not uniform, and evidence in favour of support strategies is lacking.

Previous systematic reviews have examined RTW outcomes in individuals with LC. For instance, Gualano *et al* specifically reviewed RTW outcomes in previously hospitalised COVID-19 patients, highlighting how persistent symptoms and country-specific factors affected the likelihood of RTW, and emphasised the role of occupational physicians in adapting duties.^12^ Ottiger *et al* conducted a systematic review and meta-analysis, estimating a pooled RTW rate of approximately 61% and emphasising that many individuals require job modifications.^13^ Their findings highlight the physical and psychological barriers to full RTW and point to the importance of accommodations, supportive policies and occupational rehabilitation.

Building on prior work, this rapid review took a broader and more integrated approach by examining both workplace- and non-workplace-based support for RTW. We specifically explored the barriers and facilitators to effective support for RTW, alongside individual strategies employed by PwLC to facilitate their RTW. Additionally, we considered the enabling or limiting influence of organisational policies, including financial and structural support mechanisms provided by employers.

## Methods

A review protocol was prepublished on PROSPERO (ID: CRD42023478126) and reported in line with the Preferred Reporting Items for Systematic Reviews and Meta-Analyses-Rapid Reviews (PRISMA-RR) with guidelines and adjustments made to accommodate qualitative research.^14^

### Eligibility criteria

Studies describing support available for RTW for PwLC were included. LC was defined as signs and symptoms developing after acute COVID-19 infection and persisting for more than 12 weeks.^1^ RTW was defined inclusively to capture a range of employment outcomes, including full or partial return to previous roles, modified duties, reduced working hours, temporary contracts and self-employment.

Eligible studies included (1) people of working age (>16 years old), (2) with self-reported or diagnosed LC symptoms lasting >12 weeks and (3) published in English. Studies of people who never had COVID-19 or with conditions overlapping with (but not including) LC (eg, chronic fatigue, postural orthostatic tachycardia syndrome) were excluded.

Studies investigated (1) non-workplace based support for RTW for PwLC; (2) workplace-based support for RTW for PwLC; and (3) barriers and facilitators for those with LC to RTW.

### Data sources

We searched MEDLINE, Embase, American Psychological Association PsycINFO, evidence-based medicine reviews (including the Cochrane Central Register of Controlled Trials and Cochrane Database of Systematic Reviews), Web of Science, Google Scholar and the Health Management Information Consortium. Reference lists of relevant studies including literature reviews were hand searched for additional articles. All searches were completed in June 2024.

### Search strategy

Searches were conducted by the primary researchers (SD and HW) using synonyms for ‘long COVID’ and ‘’ joined by AND operator. The search strategy for LC included the terms ‘post covid’, ‘long covid’, ‘chronic covid’ and ‘post-acute covid’. For RTW, we used ‘return to work’, ‘sickness absence’, ‘employment’, ‘vocational rehabilitation’, ‘occupational rehabilitation’, ‘back to work’, ‘work participation’, ‘workability’, ‘work ability’, ‘sick leave’, ‘illness day*’, ‘disability leave’ and ‘absenteeism’. Preliminary searches were finalised in discussions with RW, MvT, DM, DF, AC and DB. The full search strategy can be found in online supplemental material 1.

### Selection of studies

After removal of duplicates, studies underwent abstract and title screening against eligibility criteria by two researchers (SD and HW). Dual screening was performed for a random sample of 20% of the records to ensure consistency. The remaining records were abstract and title screened independently by one researcher. Dual full-text screening of a random sample of 20% of records was conducted to obtain relevant studies. The remaining records were full text screened independently by one researcher. Differences were discussed and reconciled with input from a third author (RW) if required.

### Data extraction and quality assessment

Data from the final set of studies was extracted into an agreed proforma in Excel. The data extraction table was piloted by two researchers (SD and HW) using three studies and revised accordingly. Data extracted included study characteristics (eg, aim, design, country), study population (eg, demographics including age and gender, employment status, sample size, duration of LC symptoms), characteristics of interventions and comparators (eg, type of intervention, treatment duration, patient work-related quantitative outcomes) and qualitative results (eg, type of workplace support, providers of support if reported, barriers and facilitators to support, individual strategies for RTW).

Study quality was evaluated using the Critical Appraisal tools for use in JBI Systematic Reviews.^15^ The following JBI checklists were applied based on study design: qualitative research, cross-sectional studies, case reports, randomised controlled trials (RCTs), cohort studies and quasi-experimental studies. The checklists include subscale items that were evaluated with ‘yes’, ‘no’ or ‘unclear’. Risk of bias was described as low, moderate or high quality based on the tool. Any disagreements were resolved by discussion or by involving another author (RW).

### Data synthesis

A narrative synthesis was performed to present both quantitative and qualitative results. A meta-analysis was not feasible due to substantial heterogeneity in study designs, intervention types and outcome measures related to RTW. As outlined in the PRISMA-RR guidelines, narrative synthesis is appropriate in cases where variation across studies precludes statistical pooling.^14^

Interventions were categorised as workplace-based or non-workplace-based support programmes with quantitative work-related outcomes. We created summary tables to explore the clinical context, intervention components and the impact on patients’ RTW and other workplace outcomes. Results from qualitative studies examining RTW support for PwLC were summarised into overarching themes related to barriers and facilitators of support to RTW, which were further divided into organisational and individual factors.

### Patient and public involvement

Scoping questions and research priorities were co-developed with a local LC patient support group and a team member with lived experience (DF).

## Results

### Search results

**Figure 1:**
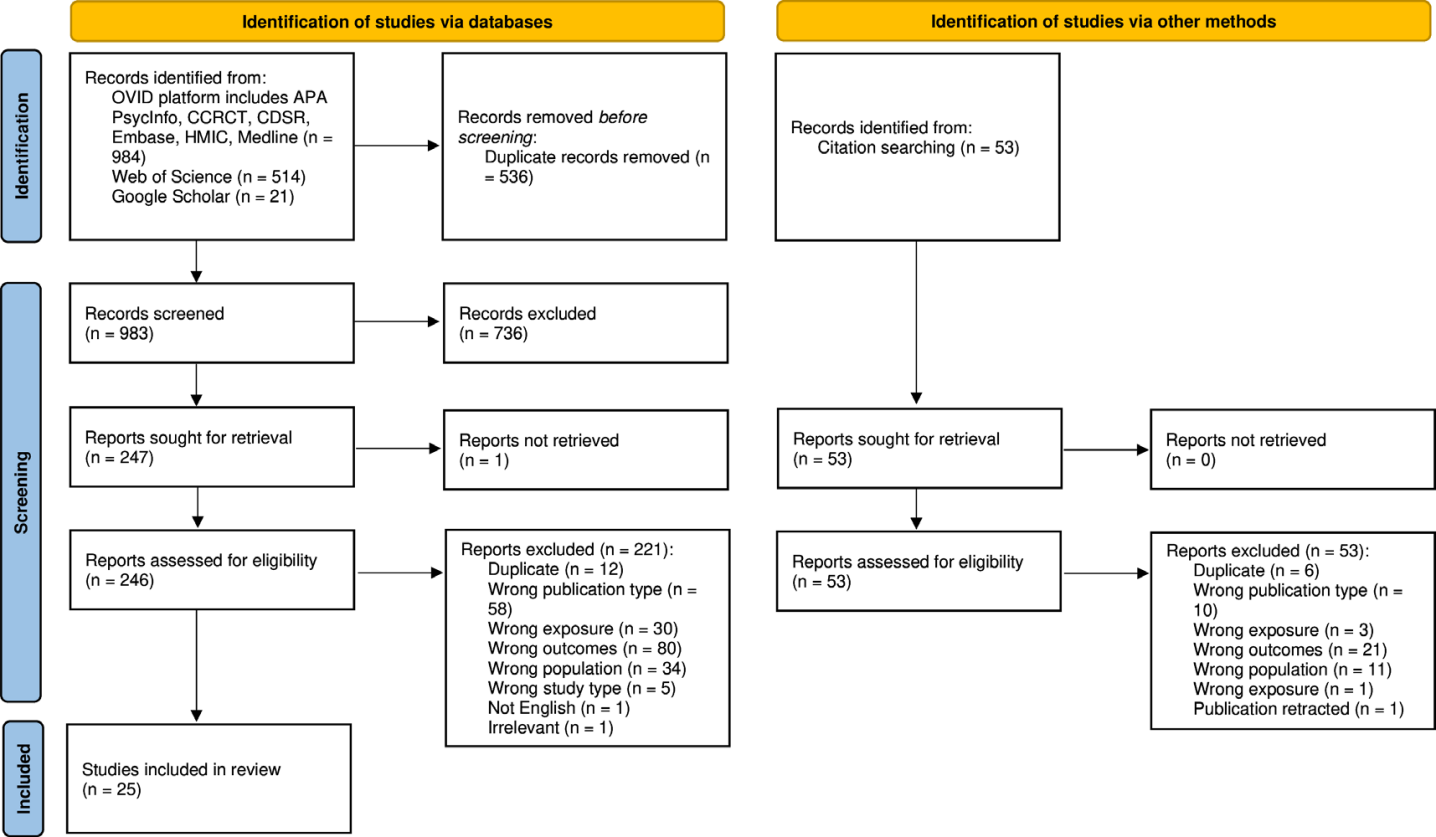
PRISMA flow diagram. APA, American Psychological Association; CCRCT, Cochrane Central Register of Controlled Trials; CDSR, Cochrane Database of Systematic Reviews; HMIC, PRISMA, Preferred Reporting Items for Systematic Reviews and Meta-Analyses.

We identified 1036 records after deduplication: 983 from the database search and 53 from citation screening. Following title and abstract screening, 299 articles underwent full-text screening and 25 met eligibility criteria for inclusion (figure 1).

### Study characteristics

Characteristics of the included studies are presented in online supplemental material 2. Of the 25 studies, two were cross-sectional,^16 17^ five were prospective cohort studies,^18^,^22^ three were retrospective cohort studies,^23^,^25^ one was a quasi-experimental study because it assessed the effects of an intervention on participants without randomisation,^26^ two were case studies,^27 28^ 10 were qualitative studies,^1029^,^37^ and two were RCTs.^38 39^ All studies were published between 2022–2024 and predominantly conducted in Europe (n=17) and North America (n=6), one in Asia and one was multinational.

The studies included 2964 participants, ranging from 50% and 100% female and aged 18 to >75 years old. Where ethnicity was reported, the majority included mostly white ethnic groups (ranging from 68% to 87% white ethnic groups in four studies).^29 33 34 36^

### Quality assessment

The assessed studies were heterogenous in design and applicability to the general population. Fifteen of the studies were identified as low risk of bias based on clear methodological descriptions and reliable outcome reporting. However, some of these were case studies or quasi-experimental in design, approaches inherently susceptible to selection bias. Their overall low-risk ratings nonetheless reflect strong internal validity within the scope of their design limitations, as assessed using JBI criteria (online supplemental material 2).

The two case studies were assessed as low risk of bias relative to the methodological standards of their respective designs.^27 28^ Both studies clearly described the participant demographics and clinical history, and the rehabilitation methods were well described. The two RCTs had a medium risk of bias due to selection and allocation issues, with non-concealed allocation in one study and unclear blinding in both and a lack of baseline similarity in group characteristics at the start of both studies.^38 39^ Among the cohort studies, four were low risk,^20 21 23 25^ three medium^18 19 22^ and one high risk.^24^ All studies had one or more limitations, including unclear LC ascertainment, lack of confounder control, unreported or unclear measurement tool validity and unaddressed incomplete follow-up. The quasi-experimental cohort study was low risk but faced limited compatibility due to the absence of a matched control group, though they used reliable pre- and post-intervention measurements with minimal participant loss to follow-up.^26^ The two cross-sectional studies were considered low risk, but one did not explicitly mention specific strategies to deal with confounding factors,^17^ and the other study had unclear measurement tool validity.^16^ Among the qualitative studies, seven were low risk,^10 29 30 32 33 36 37^ two medium^34 35^ and one high risk.^31^ Most studies showed congruity between the research question, methodology and philosophical perspective, except for Kohn *et al*,^31^ which did not adequately represent participant voices. Only two studies considered the researchers’ potential influence on the research.^10 33^

### Workplace support for return to work (RTW) with long COVID (LC) and barriers and facilitators to support

Thirteen studies reported on workplace-based support for RTW with LC^1016 17 27 29^,^37^ (online supplemental material 3). The findings were primarily based on qualitative data collected through methods such as focus groups, interviews, case studies or open-ended survey questions. The studies gathered data from the perspectives of PwLC, focusing on their lived experiences and expectations, including suggestions for support. The suggestions were made for general conditions of LC without specifying symptoms. One study used a cross-sectional design to provide quantitative survey data on how many PwLC found workplace support to be helpful or not.^17^

Commonly described workplace support measures included adjusting work arrangements, such as reducing or offering flexible work hours, allowing breaks, enabling working from home (WFH) or teleworking, adjusting job content or roles and sharing workloads. Emotional support was reported, such as good communication, regular check-ins and demonstrating understanding toward employees’ needs.^10 29 30 34^ Financial support was also reported, such as where a condition was acquired at work, through compensation claims, typically provided by employers or their insurance carriers, as well as government financial support.^33 37^ Some participants proposed vocational integration programmes as a practical support to help individuals reintegrate into the workplace after a period of absence.^32^

We categorised ‘organisational barriers’ as barriers to the provision of workplace support for RTW placed on the PwLC by the organisation, rather than by the individual. Organisational barriers to the provision of support included the lack of recognition, understanding or knowledge about LC and related symptoms.^1029^,^31 33 34 36 37^ This resulted in reports of workplace stigma associated with LC.^29^ Additionally, flexibility was sometimes not provided due to workplace constraints.^16^

Workplace culture emerged as a major concern, with organisational barriers stemming from management and colleague perceptions of participants’ ability to work. For example, a lack of understanding about the participants’ illness could lead to friction and hostility^37^ and insensitive attitudes from coworkers.^35^ Participants described a toxic environment where colleagues showed little interest in understanding the LC condition, suspicion about avoiding work and alienation.^10 29 35^ Participants also described pressure from management to ignore symptoms^10^ and questioning the legitimacy of COVID-19 health consequences.^32^

We categorised ‘individual barriers’ as emotional and financial stressors that limit the provision of workplace support for RTW in PwLC. Some participants faced individual barriers such as strain, guilt, anxiety, negative self-perception regarding their work abilities and identity crises.^10 29 31 32 35 37^ Work adjustments, such as reduced hours or medical leave, were associated with income loss, posing a significant concern and, thus, an individual barrier to obtaining such support.^10 31 32^ When WFH, some individuals were concerned about limited or no contact with colleagues and the difficulties in establishing new connections.^32^

Organisational facilitators to the provision of support included recognising LC as a legitimate condition, fostering empathy among colleagues and management and providing access to disability benefits.^10 29 31 35^ Organisational policies addressing the needs of PwLC, education initiatives to equip staff with knowledge on how to support them and workplace support groups to promote advocacy within organisations were also highlighted.^29 35^ Additionally, occupational health services recognising individual needs and capabilities were also considered a facilitator to gaining support.^35^

Straßburger *et al*^17^ surveyed organisational support for RTW with LC, finding that structural changes—specifically reduced working hours, workplace adjustments and task modifications—were considered helpful to facilitate successful reintegration into work-life, while occupational reintegration plans, health courses, general consultation and job coaching were less helpful.

Tan and Koh^27^ reported a case study on a participant who developed their own rehabilitation programme due to the lack of occupational health services provided by their workplace. Other individual strategies included finding a good balance between activity and rest,^30^ adopting a pragmatic approach to symptom management by not hiding the symptoms,^29^ communication strategies to manage expectations,^35^ knowing one’s employment rights^35^ and redefining occupational identity to find self-worth.^10^

### Non-workplace-based support for return to work (RTW) with long COVID (LC)

Twelve studies investigated non-workplace-based support for PwLC to RTW, including clinical interventions, rehabilitation programmes and multidisciplinary programmes.^18^,^2628 38 39^ Most of the intervention studies used longitudinal or experimental designs, except for Wagner *et al*,^28^ a single participant case report. Generally, interventions were effective, though Müller *et al*^21^ and Kerling *et al*^39^ found no significant differences between groups.

Several studies used the work ability index (WAI) to assess outcomes.^21 28 38 39^ The WAI is a validated self-assessment tool that measures a worker’s ability to meet the demands of their job, considering their physical and mental health, and resources.^40^ In the included studies, the WAI was used to assess changes in work readiness and functional capacity in response to various interventions.^21 28 38 39^ The results are summarised in online supplemental material 4.

Most of the intervention programmes supported all PwLC with a variety of symptoms, although one targeted neurocognitive impairments (ie, brain fog)^24^ and two targeted fatigue.^20 28^

Derksen *et al*^38^ evaluated an RCT of an interdisciplinary healthcare facilitation programme, including medical internet support from personal pilots and digital interventions. The primary focus of the intervention was to address a broader set of clinical goals, including symptom reduction and enhanced social participation. RTW outcomes were part of the evaluation, specifically through the assessment of work ability. They divided participants into three groups: an intervention group (IG), an active control group (ACG) and a comparison group (CompG), which received standard care without specific treatment. Both the IG and ACG received professional personal pilot support and digital interventions via a medical internet aid, while the IG also underwent a three-day diagnostic assessment at a specialised neurological rehabilitation clinic. Outcomes were assessed at five points: initial screening (T1), four weeks (T2), six weeks of intervention (T3, IG only), post-intervention (10–12 weeks, T4) and six weeks post-intervention (T5). The WAI scores improved for both IG and ACG at T4 and T5 compared with T2, with CompG showing slight improvement at T4. Both IG and ACG demonstrated higher work ability than CompG, although no significant difference was observed between IG and ACG over time.

LeGoff *et al*^24^ carried out a neurocognitive screening evaluation (NCSE) to assess symptoms and disabilities associated with post–COVID-19 condition and facilitating employee recovery and RTW. The NCSE functioned as both a diagnostic tool and a therapeutic intervention, providing objective feedback that may have reassured employees about their recovery potential. Its aim was to help streamline treatment recommendations and RTW decisions, leading to faster recovery timelines. Following the NCSE, the loss of workdays significantly decreased compared with pre-referral levels.^24^ The NCSE, adapted from a previous study on postconcussion cases,^41^ served both as an assessment and an intervention by providing objective feedback to patients. Frisk *et al*^26^ reported on a clinical intervention that included a phase for integrating rehabilitation changes into daily life, leading to a 20% reduction in sick leave among employed participants after three months. Tanguay *et al*^19^ reported that a telerehabilitation programme utilising pacing strategies and occupational therapy reduced the number of participants on medical leave by week eight and increased the number of participants returning to full-time work.

Altmann *et al*^18^ examined a rehabilitation programme that incorporated multimodal respiratory therapy, endurance and resistance muscle training, psychological support and educational interventions, tailored for COVID-19 and LC patients. The study found that at discharge, a higher proportion of LC participants (infected around 10 months prior) were rated as fit for work immediately compared with COVID-19 participants (infected two months prior). However, more COVID-19 participants were expected to RTW within six months. This suggests that LC participants can eventually reach a fitness level comparable to or better than recent COVID-19 participants, but there is a longer recovery period observed in some LC participants. Altmann *et al* also reported that for LC participants, difficulties in RTW were primarily due to neurocognitive pathology rather than pulmonary impairment.^18^

Brehon *et al*^23^ focused on workers who filed LC compensation claims due to workplace COVID-19 exposure. Their multidisciplinary programme, which included various therapies and psychoeducation, effectively improved RTW rates at discharge. Of those returning, 7% resumed regular duties, while 93% returned to modified duties.^23^ In addition, those who reported having modified duties available at their workplace were 3.38 times more likely to RTW on programme discharge compared with those without such options.^23^

Ghali *et al*^25^ followed patients with PCS adhering to pacing strategies for recovery. Patients who adhered more strictly to pacing strategies (ie, high engagement in pacing subscale scores) experienced higher recovery rates, defined as the ability to RTW.

Kerling *et al*^39^ evaluated a three-month home exercise plan with moderate and intense activities using the WAI. The intervention showed no change in work ability from baseline.

Kvale *et al*^22^ compared a microchoice-based rehabilitation across three groups: low back pain, LC and type 2 diabetes. The programme involved three phases: (1) preparing for change, (2) a concentrated three–four day intervention and (3) integrating changes into daily life. Patients were taught and practised how to monitor and target seemingly insignificant everyday micro-choices to break behavioural patterns that reduced functionality or contributed to health problems. Functioning improved for low back pain and LC patients, but not for diabetes patients, as measured by the Work and Social Adjustment Scale.

Schmid *et al*^20^ reported on a 14-day multimodal integrative inpatient programme, offering personalised therapies including vitamins, hydrotherapy, thermotherapy and mind-body medicine. Self-reported work ability significantly increased after treatment and six months later.

## Discussion

This rapid review sought to address the key questions: what support, if any, is being provided that assists PwLC RTW and what barriers and facilitators exist to accessing this support.

### Workplace support for return to work (RTW) with long COVID (LC) and barriers and facilitators to support

Workplace flexibility and adaptability, understanding, compassion and communication were identified as important in facilitating RTW for PwLC. The included qualitative studies described support for RTW such as reducing work hours, flexible scheduling, WFH, altering job content or roles, good communication, and emotional and financial support.^16 29 30 32 33^ These suggestions align with current UK guidance for LC which recommends that employers maintain communication and provide flexible work arrangements, such as phased returns and task adjustments, with a focus on creating a supportive workplace culture to accommodate fluctuating symptoms.^42 43^ Good working relationships, particularly between line managers and individuals, have been highlighted as crucial in enabling RTW for individuals with health problems.^44 45^ UK Government financial support includes eligibility for benefits like Statutory Sick Pay and Access to Work.^46^ These suggestions also align with those recommended for supporting workers with long-term conditions experiencing similar symptoms such as chronic fatigue syndrome.^47 48^

Included studies investigating the lived experiences of LC workers consistently reported that lack of understanding about LC symptoms, insufficient guidance or knowledge, lack of education for managers and workplaces, and workplace stigma presented major barriers to the provision of good quality RTW support.^29^,^3133^ These findings support existing research on experiences of LC.^49^ However, it is important to recognise that these perceptions, derived from qualitative research, may be subject to biases including recall bias, social desirability and selection bias. Therefore, while rich in context, the facilitators and barriers identified should be interpreted cautiously and complemented by quantitative or mixed-method studies to strengthen evidence. Moreover, while these workplace factors were consistently described, their direct impact on measurable RTW outcomes, such as timing or sustainability, was not assessed in the included studies and remains unclear. This represents a key gap in the evidence base.

Damant *et al*^50^ highlight the complex role of stigma related to LC, showing it is closely linked to symptom burden, which can exacerbate challenges in the workplace. Workplace adjustments, while necessary, may come with unintended consequences such as loss of income if working hours are reduced,^31 32^ or feelings of disconnectedness when WFH.^32^ This demonstrates the need for carefully balancing accommodations with potential impacts on workers’ well-being. Structural workplace changes, like modifying the physical environment or job tasks, appear to be more helpful for PwLC than solely educational or supportive measures, though the underlying reasons for this remain unclear.^17^ However, other studies have shown that job coaching and psychoeducational interventions can effectively help manage chronic conditions by improving perceptions of working capacity, self-efficacy and fatigue,^51^,^53^ suggesting that a combination of practical and psychosocial supports may be most effective in addressing the multifaceted challenges faced by workers with LC.

The heterogeneity of LC symptoms and their unpredictable nature make it challenging to develop universally effective support for RTW.^11^ Findings from this review demonstrate that some individuals develop their own individual coping strategies, such as balancing activity and rest, reorganising home responsibilities and openly managing symptoms. Varying and non-specific symptoms in other conditions have been effectively managed through education and strategies for self-management and mindfulness interventions.^54^,^56^ This review suggests a need for the development of and evaluation of targeted self-management programmes to facilitate RTW for those affected by LC. This should be balanced with the responsibility of employers to make workplace accommodations.

### Non-workplace-based support for return to work (RTW) with long COVID (LC)

Our findings have identified several interventions and rehabilitation programmes supporting PwLC in RTW. These include an interdisciplinary healthcare facilitation programme with digital technology support, clinical interventions that integrate improvements into daily living, multidisciplinary programmes combining therapy and medical interventions, and rehabilitation programmes offering guidance on pacing and breathing strategies.^18^,^2023 26 38^

While most interventions improved RTW outcomes, Kerling *et al*^39^ noted no significant WAI improvement following a home exercise plan, highlighting the limitations of low-intensity interventions that lack professional oversight. Motivation and adherence could play a significant role as such exercises can become monotonous and challenging, especially for PwLC experiencing fatigue, post-exertional malaise or mood disturbances.^57^,^59^

Derksen *et al*^38^ and Brehon *et al*^23^ highlight the complexity of measuring RTW outcomes in PwLC, emphasising that improvements in work ability may not be immediately apparent following interventions. The studies suggest that longer follow-up periods are necessary to fully capture the benefits of tailored rehabilitation programmes, particularly given the fluctuating and prolonged nature of LC symptoms. Furthermore, Brehon *et al*^23^ demonstrated the critical role of workplace adjustments, such as modified duties, in supporting successful RTW alongside clinical rehabilitation. These findings indicate that effective RTW support for PwLC requires a combined approach of medical intervention and adaptable workplace policies, with outcome assessments planned over extended timelines to reflect the condition’s variability.

Globally, definitions, descriptions and solutions for LC vary.^60^ The 2024 ‘National Academies of Sciences, Engineering and Medicine Long COVID Definition’ aimed to address these issues by providing a comprehensive framework that includes a wide range of symptoms and conditions.^61^ Definitions prior to this time were less established, impacting comparison and applicability of studies done in this period. Ambiguity around definitions of LC has influenced policy, legal and regulatory responses and may have altered individuals’ workplace rights. In the UK, for example, LC is classified as a disability under the Equality Act only if it has ‘substantial impact on a person’s life’ or is expected to last 12 months or more.^62^ As a result, employers may not be legally obligated to provide reasonable adjustments for individuals with LC. This may lead to different treatment for PwLC in attempted RTW and contribute to differing outcomes of successful RTW in studies.

The majority of the included intervention studies in this review were funded by compensation or insurance providers in high-income countries, for example, Germany, Canada and the USA.^18 19 21 23 24 38^ In these countries, workplace acquired COVID-19 infection and LC are treated as occupational diseases or work-related accidents and hence compensable.^63^,^66^ This recognition as a compensable condition may provide a financial incentive for insurance providers to invest in research and intervention development. The involvement of insurance providers in funding LC interventions could drive significant advancements in understanding and managing the condition.

However, there are significant equity concerns. It may lead to a focus on cost-effective solutions that prioritise short-term work-related outcomes over comprehensive long-term care. Additionally, these schemes require contributions from workers, employers and sometimes the government, making them inaccessible to those who cannot afford to pay into them. If someone has lost their job, they may not benefit from these schemes. Furthermore, these schemes are not universal and may not be available in all countries. Moreover, many of these interventions were delivered within healthcare settings. Persons with disabilities can face barriers to accessing healthcare interventions due to financial constraints, lack of health insurance, geographical limitations or demanding work schedules, making it difficult for them to attend regular appointments, among many other reasons.^67^ Therefore, they are unlikely to help everyone with LC to RTW, indicating a need for complementary provisions.

### Implications for research and policy

Symptoms of LC are not universal to all patients, multi-system, not readily visible and varied among individuals (eg, a person may experience predominantly neurological or respiratory symptoms) and over time. The unpredictability of day-to-day symptoms and differences in recovery trajectories makes a ‘one size fits all’ approach to RTW workplace support and interventions challenging. Indeed, this is true for workplace support for other chronic or recurring conditions.^68^

Our findings demonstrate that a successful RTW for PwLC requires a multidisciplinary approach involving managers, human resources, health professionals, disability support and compensation systems and a critical role for occupational health in coordinating these efforts.^69^ A tailored, long-term and flexible RTW plan is essential to accommodate the complex nature of the condition. Unfortunately, only half of the UK population has access to occupational health services, and work adjustments are not universally available or always feasible.^70^ For example, workers in small and medium-sized enterprises (SMEs) generally have less access to occupational health services.^71^ SMEs are also more reluctant to invest the time and resources into making the necessary adjustments^71^ which could lead to more job insecurity and pay differences. As a result, PwLC may experience significant barriers to necessary support to RTW and maintain their employment.

The insights gained from this review could inform policy development to help workers to return to their roles, either partially or fully, more effectively. The studies collectively affirm the effectiveness of tailored, multidisciplinary strategies in improving RTW outcomes for individuals with LC compared with less integrative approaches. The research highlights the importance of workplace adjustments, such as modified duties, and calls for scalable, evidence-based solutions that combine individualised interventions, supportive workplace environments and ongoing evaluation. These findings may also apply to managing other chronic conditions with similar symptomology, such as chronic fatigue syndrome. There may also be valuable lessons to be learnt from these conditions too, which could inform strategies for supporting PwLC.

This review focused on both non-workplace and workplace-based support for RTW with LC. We found that non-workplace-based support tended to be reported by quantitative clinical studies with effectiveness considered, while support provided by the workplace was mostly reported by qualitative studies where evaluation of effectiveness was more difficult. Similar to other long-term conditions, workplace support is much needed for returning to or remaining at work.^7 31^ Evaluating complex interventions outside of clinical settings remains a challenge for public health researchers.^72^

### Strengths and limitations

This review provides a comprehensive report of workplace- and non-workplace-based support for RTW with LC. The data extracted cover clinical interventions, multidisciplinary rehabilitation programmes, workplace support and self-developed individual support programmes. It benefits from a comprehensive literature search across major electronic databases and adheres to the rigorous reporting standards of the PRISMA guidelines.

However, the search was limited to English-language studies, which may have excluded relevant research conducted in other languages, introducing potential cultural or regional bias. In addition, studies from low- and middle-income countries may have been underrepresented due to language limitations or lower likelihood of publication in indexed English-language journals. Furthermore, our focus on work-related outcomes means that we did not include alternative support for LC patients where there may have been an impact on RTW.

Our review offers a distinctive contribution to the existing literature by exploring dimensions that have been underexamined in prior systematic reviews. In particular, we examined the role of employer-provided healthcare and compensation schemes in shaping access to and uptake of RTW support. This focus on organisational policy mechanisms provides important insight into structural facilitators or barriers that may influence RTW outcomes. Furthermore, our review incorporated evidence on individual strategies employed by PwLC to manage their symptoms and facilitate RTW. This integrated approach offers a more holistic understanding of the factors affecting successful work reintegration for PwLC.

While many studies were assessed as low risk of bias, several still had inherent limitations, particularly case studies and quasi-experimental designs, which are prone to selection bias. Many of the non-workplace based studies did not sufficiently address potential confounders, limiting the ability to attribute work outcomes solely to the intervention or other factors. For example, differences in baseline health status, comorbidities or vaccination status that may have affected recovery trajectories.^18^,^2022^ Also, some studies employed outcome measures without clearly indicating whether they used validated tools to assess LC-related RTW or work ability status.^20 24 25^ This inconsistency raises concerns about the accuracy and interpretation of findings.

National responses to LC vary significantly. Countries like the UK and USA have recognised LC as a disability under employment law, offering legal protections and workplace accommodations.^73^ Several nations, including Japan, Australia and China, have established specialist clinics and community-based services to support recovery. Additionally, substantial government funding in countries such as Canada, Germany, the UK, Japan and the USA has supported research and the development of care infrastructure.^73^ These international differences limit the generalisability of findings across national contexts.

The strict definition of LC precluded other studies that focused on conditions overlapping with LC symptoms, for example, chronic fatigue or neurocognitive conditions. Learning on barriers and facilitators to RTW in these conditions may also be relevant to those with LC. Additionally, LC definitions and access to support have changed considerably since it was recognised in mid-2020. While we acknowledge that pandemic phases and timing of diagnosis (eg, by wave or year) may influence RTW outcomes, stratified analyses by time period were not feasible due to inconsistent reporting of diagnosis dates, wave classification and timing of intervention across the included studies.

## Conclusion

Non-workplace-based interventions, such as interdisciplinary healthcare programmes and rehabilitation focusing on pacing and breathing strategies, were often funded by compensation and insurance schemes. Workplace-based support, primarily explored through qualitative studies, limited comparative evaluation of effectiveness but provided rich insight into lived experiences and workplace dynamics. These studies revealed significant organisational barriers, including a lack of understanding of LC symptoms, inadequate workplace guidance and limited manager education. Key facilitators included acknowledging and validating LC symptoms and eligibility for disability benefits.

The heterogeneity and unpredictability of LC symptoms pose significant challenges for supporting working age individuals. Further research is needed to uncover the specific RTW needs of PwLC, while addressing barriers and leveraging facilitators. Establishing consistent guidelines on LC’s definition, compensation and disability status could enhance support efforts and guide intervention development. Acknowledging RTW as a key health outcome and consistently documenting it in research on PwLC is crucial for driving progress in this field.

## Supplementary material

10.1136/bmjopen-2025-101698 online supplemental file 1

10.1136/bmjopen-2025-101698 online supplemental file 2

10.1136/bmjopen-2025-101698 online supplemental file 3

10.1136/bmjopen-2025-101698 online supplemental file 4

## Data Availability

Data are available upon reasonable request.
