# Supplementary material for: Return to work with long COVID: a rapid review of support and challenges
Source: BMJ Open. 2025 Oct 7;15(10):e101698. doi: 10.1136/bmjopen-2025-101698 (PMC12506103; doi:10.1136/bmjopen-2025-101698)
Supplement: Supplementary file 5 [file bmjopen-2025-101698.reviewer_comments.pdf]

PEER REVIEW HISTORY

BMJ Open publishes all reviews undertaken for accepted manuscripts. Reviewers are asked to complete a checklist review form and are provided with free text boxes to elaborate on their assessment. These free text comments are reproduced below.

ARTICLE DETAILS

Title (Provisional)

Return to Work with Long COVID: A Rapid Review of Support and Challenges

Authors

Daniels, Sarah; Wei, Hua; McElvenny, Damien M; van Tongeren, Martie; Bramwell, Donna; Coleman, Anna; Forde, Davine; Wiggans, Ruth

---

VERSION 1 - REVIEW

**Reviewer**	**1**
**Name**	**Ferraro, Francesco**
**Affiliation**	**University of Derby, Clinical Exercise and Rehabilitation Research Centre**
**Date**	**25-Apr-2025**
**COI**	**None**

Thank you for completing the submission of the manuscript titled "Return to Work with Long COVID: A Rapid Review of Support and Challenges" to BMJ Open. I believe the document is very well presented and makes a valuable contribution to the field. It should be considered for publication, pending minor revisions. Please refer to the following considerations to strengthen the quality and clarity of your work.

Abstract

Page 2, Lines 17–20: The objective is clear, but the methods section could be more concise. Consider summarising the search scope and focusing on the most critical inclusion criteria.

Page 2, Line 50: The phrase “and address potential barriers and facilitators to effective support” is vague. Please clarify what constitutes "effective support"—whether this refers to practical interventions, policy frameworks, or other mechanisms.

Introduction

Page 3, Line 31: The phrasing “LC is varyingly experienced, defined and understood…” is slightly awkward. A suggested revision is: “LC is variably experienced, inconsistently defined, and differently understood…”.

Page 3, Line 34: Typo — “may have had difference experiences” should read “different experiences.”

I recommend expanding on the broader societal and economic impacts of Long COVID to better frame the rationale for your study. This would help convey the urgency and relevance of your work. Consider integrating recent work such as:

Faghy, M. A. et al. (2022). Is long COVID the next global health crisis? Journal of Global Health, 12, 03067.

Additionally, the introduction would benefit from stronger engagement with prior literature focused on Long COVID. At present, it lacks a comprehensive acknowledgement of existing evidence, particularly studies involving similar cohorts. Strengthening this section will provide a clearer “so what” justification for the review and clarify the novelty of your approach.

Methods

Page 4, Lines 26–30: The definition of RTW is quite broad. Please clarify whether this includes modified duties, temporary contracts, or self-employment.

Page 5, Lines 16–18: When referring to the JBI tools, specify which checklist(s) were used (e.g. for qualitative studies, RCTs, cohort studies), to enhance transparency and replicability.

Page 5, Line 54: Clarify that meta-analysis was not feasible due to heterogeneity in intervention types and outcome measures. Consider citing PRISMA-RR guidelines regarding conditions under which narrative synthesis is appropriate.

Results

Page 6, Lines 26–28: The sentence “although a number of studies categorised as low risk were case studies or quasi-experimental…” could be misleading. Clarify why these were still rated low risk despite the design constraints.

Page 6, Line 40: The phrase “risk of bias within their own study design” is ambiguous. Consider rewording as: “low risk of bias relative to the methodological standards of their respective designs.”

Page 7, Line 55: Typo — “thus, and individual barrier…” should be “thus, an individual barrier.”

Discussion

Page 10, Line 31: When referring to the UK Equality Act, it would be helpful to include a citation or footnote for readers unfamiliar with the legal framework.

Page 11, Lines 3–8: The paragraph on insurance-based intervention models is important but dense. Splitting this into two shorter paragraphs would improve readability and better differentiate between accessibility and ethical implications.

Strengths and Limitations

Page 12, Line 20: This section is balanced and well-written. However, a limitation worth emphasising is the exclusion of non-English language studies, which may have introduced cultural or regional bias.

Page 12, Line 26: You could expand on how different national contexts have reported and responded to Long COVID. Consider citing:

Ciccarelli et al. (2024). Italian Journal of Health Education...

Quaranta et al. (2023). Journal of Clinical Medicine, 12(19), 6303.

Qin et al. (2024). Lancet Regional Health – Western Pacific, 52.

Page 12, Line 32: The sentence "did not consider these different time periods" is vague. Suggest clarifying whether stratified analyses by diagnosis year or COVID wave were considered, or explaining why such an approach was not feasible.

Conclusion

Page 13, Lines 4–6: The sentence "Workplace-based support, primarily explored qualitatively…" would benefit from clarification. Specify that qualitative approaches limited comparative effectiveness evaluation, but still provided rich context on lived experiences.

**Reviewer**	**2**
**Name**	**Zhang , Joyce C**
**Affiliation**	**University of Toronto, Occupational Medicine**
**Date**	**21-Jun-2025**
**COI**	**None**

I read with great interest this rapid review on non-workplace and workplace supports for facilitating LC patients' return to work. This is a very salient topic that LC is associated with significant loss-time from the workplace and loss of productivity. As the authors have pointed out, studying this area in itself is challenging as the definitions of LC vary, as do the symptoms presentation and consequently the functional needs of the individual.

In the introduction, the authors outlined the definition of LC, its affected demographic, and the significant impact the diagnosis has on work disability. The heterogeneity of symptoms and the non-linear recovery timeframes of the condition present additional challenges for return to work, and consequently to workers and employers.

In methods, the authors adequately described their methodology in their search strategy and quality control . Search turns were reasonable, as is study selection using a standard PRISMA approach. The authors noted that owing to study variability, meta-analysis was not possible. This is reasonable given the width and breadth of intervention strategies that have been published as clinical interventions. A qualitative approach is also reasonable in assessing workplace facilitators and barriers, as very few quantitative studies exist.

In the results and discussion sections, the authors summarized the findings of the studies included and their outcomes. This is perhaps the most interesting section for insurance boards, compensation boards, and healthcare providers as it recommends interventions that have yielded positive outcomes. The WAI index, though mentioned, requires explanation and expansion for the readers, given it was cited in multiple studies. I do find that the discussion section on the non-workplace studies is slightly repetitive to the results section. I recommend the authors consider a higher level of summarizing of the overall information in the studies without going into significant details. It is also important for the authors to comment if other rapid reviews of a similar nature have been published and what this review adds compared to other reviews if they exist.

Given that the workplace factors were studied qualitatively, it is very important as well to point out that the perceptions of the facilitators and barriers are very prone to bias. A larger emphasis on this is warranted. What is lacking is the commentary on whether these workplace facilitators and barriers have impacted RTW in a meaningful way (earlier RTW). If this information is not possible, it should be explicitly discussed.

Another recommendation is for the authors to reconsider the subheadings under discussion. They should match the groups of studies looked at in the review such that it is easier for the readers to follow. For example, instead of “interventions that improve work ability of people with long COVID”, to match it to “non-workplace-based support for RTW).

Thank you for the opportunity to review this manuscript.

VERSION 1 - AUTHOR RESPONSE

Reviewer: 1

Abstract

Page 2, Lines 17–20: The objective is clear, but the methods section could be more concise. Consider summarising the search scope and focusing on the most critical inclusion criteria.

Thank you for this helpful suggestion. We have revised the methods section of the abstract to be more concise by summarising the list of databases searched and focusing on the most essential inclusion criteria (see Abstract, page 1-2). We have also reformatted the abstract to follow the structured headings as requested by the Editor (see above).

Page 2, Line 50: The phrase "and address potential barriers and facilitators to effective support" is vague. Please clarify what constitutes "effective support"—whether this refers to practical interventions, policy frameworks, or other mechanisms.

Thank you for this helpful comment. We agree that the phrase "effective support" required clarification. While our review included both workplace-based and non-workplace-based RTW support for people with long COVID, barriers and facilitators were examined specifically in relation to workplace-based support. We have therefore revised the abstract to clarify that "effective support" refers to workplace-based interventions, organisational practices, and employer-led policies that enable sustained RTW (see Abstract, page 2).

Introduction

Page 3, Line 31: The phrasing "LC is varyingly experienced, defined and understood..." is slightly awkward. A suggested revision is: "LC is variably experienced, inconsistently defined, and differently understood...".

We have revised the sentence as recommended for improved clarity and flow. The updated sentence now reads: "LC is experienced differently across individuals, inconsistently defined in the literature, and variously interpreted in practice." This change has been made on page 3, paragraph 4, of the revised manuscript.

Page 3, Line 34: Typo — "may have had difference experiences" should read "different experiences."

Thank you for spotting this. We have corrected the typo, and the phrase now reads "different experiences" as intended. This change has been made on page 3 of the revised manuscript.

I recommend expanding on the broader societal and economic impacts of Long COVID to better frame the rationale for your study. This would help convey the urgency and relevance of your work. Consider integrating recent work such as:

Faghy, M. A. et al. (2022). Is long COVID the next global health crisis? Journal of Global Health, 12, 03067.

Thank you for your feedback. We have updated the introduction to include the 2025 Delphi consensus by Ewing et al., which reports Long COVID affects over 400 million people globally and calls for clearer definitions, diagnosis, treatment, and research. This complements earlier

work by Faghy et al. (2022) on symptom complexity and healthcare challenges. We believe this improves the context and relevance of our study (see Introduction on page 3, paragraph 2).

Additionally, the introduction would benefit from stronger engagement with prior literature focused on Long COVID. At present, it lacks a comprehensive acknowledgement of existing evidence, particularly studies involving similar cohorts. Strengthening this section will provide a clearer "so what" justification for the review and clarify the novelty of your approach.

Thank you for this helpful suggestion. In response, we have revised the introduction to engage more directly with prior systematic reviews examining RTW outcomes among individuals with Long COVID, including those by Ottiger et al. (2024) and Gualano et al. (2022). These studies have significantly advanced understanding of RTW outcomes and the impact of persistent symptoms on occupational functioning.

Our review builds on this important work by offering a broader and more integrated perspective. Specifically, we include both workplace- and non-workplace-based support strategies and synthesise barriers and facilitators to RTW, incorporating evidence drawn from the lived experiences of PwLC. In addition, we examine individual strategies used by PwLC to facilitate their RTW and consider how organisational policies, including employer-provided healthcare and compensation schemes, shape access to support. These aspects are underexplored in previous literature and strengthen the practical and policy relevance of our findings.

We have revised the Introduction to reflect these distinctions more clearly (see page 4, paragraph 5 and 6) and we have also added this detail to the Strengths and Limitations section to emphasise the unique contribution of our review (see page 14, paragraph 2).

Methods

Page 4, Lines 26–30: The definition of RTW is quite broad. Please clarify whether this includes modified duties, temporary contracts, or self-employment.

We have clarified our definition of RTW in the Methods section to reflect its inclusive scope. Specifically, we now state that RTW encompasses full or partial return to previous roles, modified duties, reduced working hours, temporary contracts, and self-employment. This change is now reflected on page 4, paragraph 2 of the *Eligibility criteria* section.

Page 5, Lines 16–18: When referring to the JBI tools, specify which checklist(s) were used (e.g. for qualitative studies, RCTs, cohort studies), to enhance transparency and replicability.

We have revised the Methods section to specify which JBI Critical Appraisal Checklists were used based on the study design. These included tools for qualitative research, cross-sectional studies, case reports, RCTs, cohort studies, and quasi-experimental designs. Please see page 5, paragraph 2 of the *Data extraction and quality assessment* section.

Page 5, Line 54: Clarify that meta-analysis was not feasible due to heterogeneity in intervention types and outcome measures. Consider citing PRISMA-RR guidelines regarding conditions under which narrative synthesis is appropriate.

We have clarified in the Data Synthesis section that a meta-analysis was not feasible due to heterogeneity in intervention types, study designs, and outcome measures. We have also

referenced the PRISMA-RR guidelines to justify our use of narrative synthesis in this context. Please see page 5, paragraph 1 for the updated text.

Results

Page 6, Lines 26–28: The sentence "although a number of studies categorised as low risk were case studies or quasi-experimental..." could be misleading. Clarify why these were still rated low risk despite the design constraints.

Thank you for this observation. We have clarified this sentence to explain why certain studies with inherently weaker designs (e.g. case studies, quasi-experiments) were still rated as low risk of bias. Specifically, their classification was based on the robustness of reporting, methodological transparency, and internal validity as evaluated using the appropriate JBI checklists, despite the recognised limitations in generalisability or selection bias. This clarification has been added to the *Quality assessment* section (page 6, paragraph 1).

Page 6, Line 40: The phrase "risk of bias within their own study design" is ambiguous. Consider rewording as: "low risk of bias relative to the methodological standards of their respective designs."

Thank you for your suggestion. We have revised the phrase "risk of bias within their own study design" to "low risk of bias relative to the methodological standards of their respective designs" to improve clarity and precision (page 6, paragraph 2 of *Quality assessment*).

Page 7, Line 55: Typo — "thus, and individual barrier..." should be "thus, an individual barrier."

Thank you for catching this typo. We have corrected "thus, **and** individual barrier" to "thus, **an** individual barrier."

Discussion

Page 10, Line 31: When referring to the UK Equality Act, it would be helpful to include a citation or footnote for readers unfamiliar with the legal framework.

Thank you for the suggestion. We have added a citation for the UK Equality Act to provide readers with easy access to the legal framework referenced in the discussion (page 12, paragraph 5 of *Non-workplace-based support for RTW with LC* sub-section of the Discussion).

Page 11, Lines 3–8: The paragraph on insurance-based intervention models is important but dense. Splitting this into two shorter paragraphs would improve readability and better differentiate between accessibility and ethical implications.

Thank you for this helpful suggestion. In response, we have revised the paragraph to improve clarity and readability by dividing it into two distinct sections. The first focuses on the role of insurance-based models and their contribution to intervention development, while the second outlines concerns regarding accessibility and ethical implications. This restructuring aims to better differentiate the systemic opportunities from the potential barriers these models pose, particularly for those without access to employer- or state-based insurance schemes (see page 12-13, paragraph 5 and 6 of *Non-workplace-based support for RTW with LC* sub-section of the Discussion).

Strengths and Limitations

Page 12, Line 20: This section is balanced and well-written. However, a limitation worth emphasising is the exclusion of non-English language studies, which may have introduced cultural or regional bias.

Thank you for this helpful suggestion. We agree that limiting the search to English-language publications may have introduced language and regional bias. In response, we have revised the discussion section to acknowledge this limitation explicitly and to highlight the potential underrepresentation of studies from low- and middle-income countries (see *Strengths and limitations*, page 14, paragraph 2).

Page 12, Line 26: You could expand on how different national contexts have reported and responded to Long COVID. Consider citing:

Ciccarelli et al. (2024). Italian Journal of Health Education...

Quaranta et al. (2023). Journal of Clinical Medicine, 12(19), 6303.

Qin et al. (2024). Lancet Regional Health – Western Pacific, 52.

Thank you for this helpful suggestion. We have now expanded the relevant section to include international comparisons drawn from a recent systematic review by Song et al. (2024), which documents global policy responses to LC. This addition outlines how countries such as the UK and USA have formally recognised LC as a disability under employment law, enabling workplace accommodations. We also note how countries including Japan, Australia, and China have implemented dedicated LC clinics and support systems, while national governments in Canada, Germany, UK, Japan and the USA have invested significantly in LC research and service development. These examples highlight the variability in national responses and reinforce the point that generalisability across contexts remains limited (see page 14, paragraph 5 of *Strengths and limitations*).

Page 12, Line 32: The sentence "did not consider these different time periods" is vague. Suggest clarifying whether stratified analyses by diagnosis year or COVID wave were considered, or explaining why such an approach was not feasible.

Thank you for this helpful comment. We have revised the sentence to clarify our approach. Specifically, we now acknowledge that timing of diagnosis (e.g. by COVID wave or year) may affect RTW outcomes but explain that stratified analysis by time period was not feasible due to inconsistent reporting across included studies. Many papers did not specify diagnosis dates, wave classification, or timing of intervention, which limited our ability to analyse changes over time. The revised sentence appears on page 14 and 15, paragraph 6 of *Strengths and limitations*.

Conclusion

Page 13, Lines 4–6: The sentence "Workplace-based support, primarily explored qualitatively..." would benefit from clarification. Specify that qualitative approaches limited comparative effectiveness evaluation, but still provided rich context on lived experiences.

Thank you for this helpful suggestion. We have revised the sentence to clarify that while qualitative studies limited our ability to assess comparative effectiveness across interventions, they offered valuable insights into lived experiences and workplace dynamics. The revised text appears on page 15, paragraph 1 of the Conclusion.

<u>Reviewer: 2</u>

In the results and discussion sections, the authors summarized the findings of the studies included and their outcomes. This is perhaps the most interesting section for insurance boards, compensation boards, and healthcare providers as it recommends interventions that have yielded positive outcomes. The WAI index, though mentioned, requires explanation and expansion for the readers, given it was cited in multiple studies.

Thank you for your helpful comment. We have now expanded our description of the WAI in the Results section. Specifically, we clarify that the WAI is a validated self-assessment tool that measures a worker's ability to meet job demands, accounting for physical and mental health, and resources (Ilmarinen et al., 2007). We have also elaborated on how the WAI was applied in the included studies (page 8, paragraph 2 of the Results sub-section titled *Non-workplace-based support for RTW with LC*).

I do find that the discussion section on the non-workplace studies is slightly repetitive to the results section. I recommend the authors consider a higher level of summarizing of the overall information in the studies without going into significant details.

Thank you for your helpful feedback. We have amended paragraphs 2 and 3 in the Discussion sub-section titled *Non-workplace-based support for RTW with LC* on page 12. These paragraphs have been revised to move away from detailed reporting of individual study results and instead focus on summarising what the studies collectively show and providing a clearer discussion of their implications. This approach better highlights the complexity of measuring RTW outcomes, the importance of longer follow-up periods, and the role of workplace adjustments alongside rehabilitation efforts.

Although this was not explicitly requested by the reviewer, we identified that paragraph 3 of the *Workplace support for RTW with LC, and barriers and facilitators to support* section (page 11) also contained some repetition of study results. Based on the reviewer's helpful comment about reducing repetition, we took the opportunity to amend this paragraph as well. The revision now focuses more on discussing the implications of the findings, such as the complex role of stigma, potential unintended consequences of workplace adjustments, and the relative benefits of different types of interventions, rather than restating detailed study outcomes.

It is also important for the authors to comment if other rapid reviews of a similar nature have been published and what this review adds compared to other reviews if they exist.

We thank the reviewer for this helpful comment. In response, we have clarified how our rapid review differs from and complements previous work. While systematic reviews such as those by Ottiger et al. (2024) and Gualano et al. (2022) have provided important insights into RTW outcomes and the impact of persistent symptoms among people with Long COVID, our review adds several novel contributions.

Specifically, we adopt a broader and more integrated perspective by synthesising evidence on both workplace- and non-workplace-based support for RTW. We also include analysis of organisational factors, such as employer-provided healthcare and compensation schemes, that may influence access to support. Furthermore, we examine individual strategies used by people with Long COVID to facilitate their RTW, incorporating lived experience data where available. These aspects have received less attention in previous reviews and contribute to the practical and policy relevance of our findings.

We have now added this distinction to the Introduction (page 4, paragraph 5 and 6) and to the Strengths and Limitations section (page 14, paragraph 2).

Given that the workplace factors were studied qualitatively, it is very important as well to point out that the perceptions of the facilitators and barriers are very prone to bias. A larger emphasis on this is warranted.

Thank you. We agree that the qualitative nature of the workplace factors studies means the reported facilitators and barriers are based on subjective perceptions, which are inherently prone to bias. In response, we have expanded the Discussion to acknowledge this limitation more explicitly and emphasise the need for cautious interpretation of these findings (see page 11, paragraph 2 of *Workplace support for RTW with LC, and barriers and facilitators to support*).

What is lacking is the commentary on whether these workplace facilitators and barriers have impacted RTW in a meaningful way (earlier RTW). If this information is not possible, it should be explicitly discussed.

Thank you for this helpful comment. We agree that it is important to clarify the extent to which the workplace-related facilitators and barriers identified in the qualitative studies impacted RTW outcomes in a measurable way. We have now explicitly addressed this in the discussion section (page 11, paragraph 2, sub-section *Workplace support for RTW with LC, and barriers and facilitators to support* of the Discussion), noting that while these workplace factors were consistently described, their direct impact on RTW timing or sustainability was not assessed in the included studies. We have emphasised that this represents a key gap in the current evidence base and suggested that future research should investigate the effectiveness of workplace interventions using quantitative or mixed-method approaches.**Another recommendation is for the authors to reconsider the subheadings under discussion. They should match the groups of studies looked at in the review such that it is easier for the readers to follow. For example, instead of "interventions that improve work ability of people with long COVID", to match it to "non-workplace-based support for RTW).**

Thank you for this helpful suggestion. We agree that aligning the discussion subheadings with the groups of studies presented in the results would improve clarity and reader navigation. In response, we have revised and reordered the sub-sections in the Discussion to match the flow and structure of the Results section.

VERSION 2 - REVIEW

**Reviewer**	**1**
**Name**	**Ferraro, Francesco**
**Affiliation**	**University of Derby, Clinical Exercise and Rehabilitation Research Centre**
**Date**	**28-Aug-2025**
**COI**	

Thank you for addressing the comments and applying the required correction. Very well done.
